# Comparing Trajectory of Surgical Aortic Valve Replacement in the Early vs. Late Transcatheter Aortic Valve Replacement Era

**DOI:** 10.3389/fcvm.2021.680123

**Published:** 2021-06-22

**Authors:** Gabby Elbaz-Greener, Guy Rozen, Fabio Kusniec, Ibrahim Marai, Shemy Carasso, Dennis T. Ko, Harindra C. Wijeysundera, Ronny Alcalai, David Planer, Offer Amir

**Affiliations:** ^1^Department of Cardiology, The Faculty of Medicine, Hadassah Medical Center, Hebrew University of Jerusalem, Jerusalem, Israel; ^2^Division of Cardiovascular Medicine, Baruch Padeh Medical Center, Poria, Israel; ^3^The Azrieli Faculty of Medicine in the Galilee, Bar-Ilan University, Safed, Israel; ^4^Cardiology Division, Harvard Medical School, Massachusetts General Hospital, Boston, MA, United States; ^5^Division of Cardiology, Schulich Heart Centre, Sunnybrook Health Sciences Centre, University of Toronto, Toronto, ON, Canada; ^6^ICES, Toronto, ON, Canada; ^7^Sunnybrook Research Institute, University of Toronto, Toronto, ON, Canada; ^8^Institute for Health Policy Management and Evaluation, University of Toronto, Toronto, ON, Canada

**Keywords:** aortic valve replacement, TAVR, transcatheter and surgical aortic valve replacement, surgical aortic valve implantation, transcatheter aortic replacement, aortic stenosis

## Abstract

**Background:** Traditionally, the only effective treatment for aortic stenosis was surgical aortic valve replacement (SAVR). Transcatheter aortic valve replacement (TAVR) was approved in the United States in late 2011 and provided a critical alternative therapy. Our aims were to investigate the trends in the utilization of SAVR in the early vs. late TAVR era and to assess SAVR and TAVR outcomes.

**Methods:** Using the 2011–2017 National Inpatient Sample database, we identified hospitalizations for patients with a most responsible diagnosis of aortic stenosis during which an aortic valve replacement (AVR) was performed, either SAVR or TAVR. Patients' sociodemographic and clinical characteristics, procedure complications, length of stay, and mortality were analyzed. Multivariable analyses were performed to identify predictors of in-hospital mortality. Piecewise regression analyses were performed to assess temporal trends in SAVR and TAVR utilization.

**Results:** A total of 542,734 AVR procedures were analyzed. The utilization of SAVR was steady until 2014 with a significant downward trend in the following years 2015–2017 (*P* = 0.026). In contrast, a steady upward trend was observed in the TAVR procedure with a significant increase during the years 2015–2017 (*P* = 0.006). Higher in-hospital mortality was observed in SAVR patients. The mortality rate declined from 2011 to 2017 in a significantly higher proportion in the TAVR compared with the SAVR group.

**Conclusion:** Utilization of SAVR showed a downward trend during the late TAVR era (2015–2017), and TAVR utilization demonstrated a steady upward trend during the years 2011–2017. Higher in-hospital mortality was recorded in patients who underwent SAVR.

## Introduction

Since the first human transcatheter aortic valve replacement (TAVR) performed in 2002, TAVR has rapidly transitioned from an innovative procedure intended for compassionate use to the standard of care for elderly patients with severe symptomatic aortic stenosis (AS). Over the past two decades since Cribier's description of the first human TAVR in 2002 (1), uptake has increased exponentially across the world (2, 3). In 2004, high-surgical-risk TAVR feasibility studies were initiated, leading to the Conformité Européenne (CE) mark being granted in 2007 (2–4) followed by FDA and Health Canada approval in 2011 (3, 5). Over this period, more than 500,000 procedures have been performed in more than 70 countries (3, 6).

The indications for TAVR have evolved quickly from compassionate use as the last resort to being the preferable option for inoperable, high-risk patients (7, 8) and more recently as a reasonable alternative for intermediate-risk populations (9–11) and low-risk patients (12, 13). TAVR has evolved from a challenging intervention to a simple, efficient, and streamlined procedure that has become the standard of care (6, 14).

In view of the fact that TAVR has transitioned to the standard of care, implementation issues are increasingly important. Two conceptual models are involved in this process. The first, known as the “life cycle,” (15) describes the gradual penetration of a new product or technology over time from the development of the required threshold of robust clinical evidence to device iteration, physician training, and subsequent health system planning for dissemination (15, 16). The second is termed the “disruptive technology or innovation” and describes a new technology that substitutes an established one that shares the same market (15). This would explain TAVR penetration distributed with the surgical aortic valve replacement (SAVR) market and then replacing SAVR.

Indeed, some registries demonstrate changes in TAVR and SAVR utilization; for example, in Germany, the Applied Quality Improvement and Research in Health Care (AQUA) reports show that the annual number of SAVR procedures decreased between the years 2008 to 2014. In contrast, the number of TAVR procedures increased 20-fold in 2014 (17). Reported penetration rates in the United States and Canada have been low compared with countries in western Europe due to different regulatory requirements that delayed market access (5).

Using the National Inpatient Sample (NIS) database, our objective was to assess the trends in utilization of SAVR in the early vs. late TAVR era (2011–2014 vs. 2015–2017) and to assess SAVR and TAVR outcomes.

## Methods

### Data Collection

The data were obtained from the NIS database, the Healthcare Cost and Utilization Project (HCUP), and the Agency for Healthcare Research and Quality (AHRQ) (18). Data from the NIS data sets were deidentified, and therefore, this study was deemed exempt from institutional review by the human research committee.

The NIS is the largest collection of all-payer data on inpatient hospitalizations in the United States. The data set represents an ~20% stratified sample of all inpatient discharges from U.S. hospitals (19). This information includes patient- and hospital-level factors, such as patient demographic characteristics, primary and secondary diagnoses and procedures, AHRQ comorbidities, length of stay (LOS), hospital region, hospital teaching status, hospital bed size, and cost of hospitalization. National estimates were calculated using the patient- and hospital-level sampling weights that were provided by NIS.

For the purpose of this study, we obtained data for the years 2011 to 2017. The International Classification of Diseases, 9th and 10th Revisions, Clinical Modifications (ICD-9-CM and ICD-10-CM) were used for reporting diagnoses and procedures in the NIS database. These are standard lists of six-character alphanumeric codes to describe diagnoses in the U.S. health care system in order to improve consistency among physicians in recording patient symptoms and diagnoses for billing and clinical research purposes. ICD-9-CM and ICD-10-CM were used in the NIS database during 2011 to the last quarter of 2015 and from the last quarter of 2015–2017, respectively. For each index hospitalization, the database provides a principal discharge diagnosis and a maximum of 14 or 24 additional diagnoses (depending on the year) in addition to a maximum of 15 procedures.

We identified patients 18 years of age or older with a primary diagnosis of AS based on ICD-9-CM codes 395.0, 395.2, 396, 396.2, 746.3, and 424.1 and based on ICD-10-CM codes I35.0, I35.2, Q23.0, I06.0, I06.2, and I08.0, who underwent in-hospital SAVR or TAVR based on ICD-9-CM procedure codes for PR1-PR15. ICD-9-CM codes 35.21 and 35.22 were used for the SAVR group, and 35.05 (trans-femoral) and 35.06 (trans-apical) were used for the TAVR group. ICD-10-CM codes 02RF07Z, 02RF08Z, 02RF0JZ, and 02RF0KZ were used for the SAVR group, and 02RF37Z, 02RF38Z, 02RF3JZ, 02RF3KZ, and X2RF332 (trans-femoral), 02RF37H, 02RF38H, 02RF3JH, and 02RF3KH (trans-apical) were used for the TAVR group.

We used the Deyo-Charlson Comorbidity Index (Deyo-CCI), a modified version of the Charleson Index, which predicts the risk of death within 1 year of hospitalization for patients with specific comorbid conditions. Deyo-CCI uses the ICD-9-CM diagnosis and procedure codes, the administrative data for 17 comorbidities with differential weights of 1 to 6, to calculate the final score index, ranging from 0 to 33. The following patient demographics were collected from the database: age, sex, and race. Prior comorbidities were identified from the AHRQ. Detailed information on Deyo-CCI is provided in Table A1. Higher Deyo-CCI scores indicate a greater burden of comorbid diseases and are associated with mortality 1 year after admission (20). The Deyo-CCI index has been used extensively in studies from administrative databases with proven validity in predicting short- and long-term outcomes (21, 22).

### Outcomes

The primary outcome in this study was all-cause in-hospital mortality. The secondary outcome of interest included both in-hospital complications and LOS. In-hospital complications were defined as previously reported, known in-hospital SAVR and TAVR as well as SAVR-related complications (23, 24) as follows. (1) Pericardial complications, defined as tamponade, hemopericardium, pericarditis, and pericardiocentesis; (2) cardiac complications (during or resulting from procedure), defined as cardiac block, myocardial infarction, cardiac arrest, congestive heart failure, cardiogenic shock, and others; (3) pulmonary complications, defined as pneumothorax/hemothorax, diaphragm paralysis, postoperative respiratory failure, and other iatrogenic respiratory complications; (4) hemorrhage/hematoma complications, defined as hemorrhage/hematoma complicating a procedure, acute post-hemorrhagic anemia, and hemorrhage requiring transfusion; (5) vascular complications, defined as accidental puncture or laceration during a procedure, injury to blood vessels, arteriovenous fistula, injury to retroperitoneum, vascular complication requiring surgical repair, reopening and other vascular complications; (6) infection, defined as fever, septicemia, and postprocedural aspiration pneumonia; (7) neurological, defined as nervous system complication, unspecified, central nervous system complication, iatrogenic cerebrovascular infarction, or hemorrhage cerebrovascular effect, and transient ischemic attack; (8) diaphragmatic paralysis; (9) acute renal failure; (10) reopen and conversion to open surgery; (11) device-related mechanical complication; (12) paravalvular leak (PVL); and (13) permanent pacemaker implantation (PPM). Detailed information on all ICD-9-CM and ICD-10-CM codes used to identify in-hospital complications is summarized in Appendix Table 2. LOS was defined as the time interval in days from hospital admission to hospital discharge.

### Statistical Analysis

The chi-square (χ2) test and Wilcoxon rank sum test were used to compare categorical variables and continuous variables, respectively. The NIS provided discharge sample weights that were calculated within each sampling stratum as the ratio of discharges in the universe to discharges in the sample (25). Prior to 2012, a 20% sample from all hospitals in the United States providing long-term acute care and 100% discharge data from these hospitals were retained. Beginning in 2012, however, the NIS was redesigned to construct reciprocal information, partially 20% of discharge records from all hospitals in the sampling frame. These design changes, however, do not limit multiyear analysis. To account for these revisions while performing trend analysis, AHRQ developed new patient-level discharge trend weights for the years prior to 2012. The new trend weights (called “TRENDWT”) were intended to be used instead of the earlier NIS weights (called “DISCWT”) in years prior to 2012 while performing a multiyear analysis spanning year 2012. Utilizing the new weights resulted in improved national estimates in addition to allowing for multiyear analysis of trends.

#### Trends

Piecewise regression analyses were performed to assess temporal trends in SAVR and TAVR utilization in response to an empirical inflection point corresponding to the early vs. late TAVR era in 2014.

#### Predictors of Mortality/Complications

We generated a weighted logistic regression model using “TRENDWT” to identify independent predictors of in-hospital complications and mortality. Congruent with the HCUP NIS design, the hospital identification number was used as a random effect with patient-level factors clustered within hospital-level factors. We retained all predictor variables that were associated with our primary outcome, mortality, and secondary outcome, at least one complication, with *p* < 0.05 in our final multivariable regression model.

For LOS analysis, a *p*-value was calculated from non-parametric confidence intervals to evaluate differences between SAVR and TAVR.

For all analyses, we used SAS® software version 9.4 (SAS Institute Inc., Cary, NC.). A *p*-value < 0.05 was considered statistically significant.

## Results

Out of 109,483 unweighted hospitalizations in the NIS database during the years 2011 to 2017, we included only AS hospitalizations as described above. After implementing the weighting method, these represented an estimated total of 542,734 hospitalizations for AS in patients who underwent in-hospital AVR during the index hospitalization.

### Baseline Characteristics

In the total cohort, the majority of patients were male (61.3%) and Caucasian (79.7%), and the mean age was 72.5 ± 12 years. There were significantly older patients in the TAVR group compared with the SAVR group (80.6 + 8.2 vs. 69.4 + 11.7, respectively). TAVR was performed via trans-femoral access in 92.3% of the cases. Full demographic and clinical characteristics of the study population are presented in Table 1.

**Table 1:** Comparison of baseline characteristics of SAVR vs. TAVR during the years 2011–2017.

	**Total cohort AVR**	**SAVR total**	**TAVR total**	***P*-value**
**Population**, ***N***
Total number^a^	109,483	79,330	30,153	
Weighted total number^b^	542,734	392,087	150,647	
Age Group, years %				<0.001
18–49	4.4	6.0	0.4	
50–59	9	11.8	1.6	
60–69	21.7	26.9	8.3	
70–79	33.3	35.8	26.9	
80–89	27.6	18.8	50.4	
>90	4	0.7	12.4	
Age mean ± SD	72.5 ± 12	69.4 + 11.7	80.6 + 8.2	<0.001
Sex, male %	67,085 (61.3)	50,977 (64.3)	16,108 (53.4)	<0.001
**Race, %**				<0.001
White	79.7	78.6	82.7	
Other	20.3	21.4	17.3	
**Comorbidities, %**
Hypertension	64.6	69.4	52.3	<0.001
Hyperlipidemia	62.4	61.2	65.6	<0.001
Cerebrovascular disease	6.6	7.1	5.1	<0.001
Congestive heart failure	12.7	6.5	28.8	<0.001
Diabetes Mellitus	31.7	30.4	35.1	<0.001
Renal failure	22.1	16.8	35.9	<0.001
Chronic pulmonary disease	24	21.4	30.9	<0.001
Smoker	5.4	7.0	1.2	<0.001
Peripheral vascular disease	22.5	20.8	26.8	<0.001
Prior Ischemic Heart disease	39.2	43.7	27.6	<0.001
Prior Percutaneous Coronary Intervention	7.5	6.8	9.4	<0.001
**Prior cardiac surgery, %**	15	8.5	32.0	<0.001
^*^Isolated AV procedure	65	52	98.8	<0.001
^**^Access site Trans-Femoral	N/A	N/A	92.3%	
**Deyo-CCI, %**				<0.001
0	18.2	22.7	6.3	
1	21.2	25.0	11.2	
2 or higher	60.6	52.2	82.5	

a *Represents the number of observations in the NIS database*.

b *Represents total national estimates after applying sampling weights*.

* *Isolated AV procedure for TAVR=Not complicated with open heart surgery*.

** *7.7% Access site Trans-Apical TAVR*.

*AVR, aortic valve replacement; Deyo-CCI, Deyo-Charlson Comorbidity Index; SAVR, surgical aortic valve replacement; TAVR, transcatheter aortic valve replacement*.

Higher prevalence rates of congestive heart failure, chronic obstructive pulmonary disease, chronic renal failure, and patients with Deyo-CCI score >2 were all observed in the TAVR population compared with the SAVR population. Furthermore, patients with prior percutaneous coronary intervention and prior sternotomy were more prevalent in the TAVR group (Table 1). In the SAVR group, more than 50% had isolated aortic intervention (Table 1).

### AVR Utilization Trends

**Figure 1:**
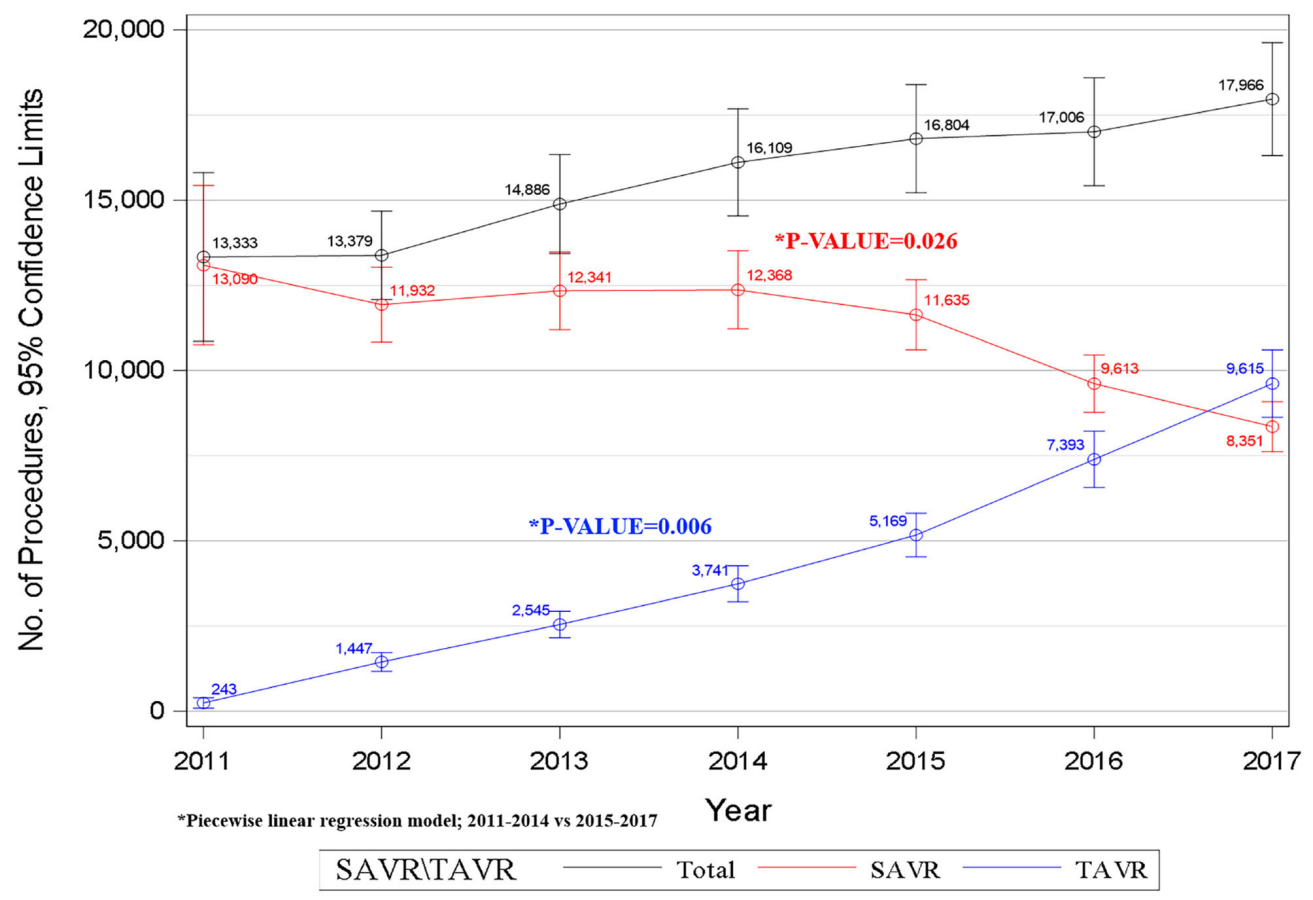
Trends in AVR procedures during the years 2011–2017.

Our data shows that the annual number of SAVR procedures decreased from 13,090 in 2011 to 8,351 in 2017. In contrast, the number of TAVR procedures increased 40-fold from 243 in 2011 to 9,615 in 2017 and has surpassed the annual number of SAVRs in 2017 (Figure 1). Using a piecewise regression analysis, we found a relatively steady utilization of SAVR in AS patients until 2014; however, following 2014, we observed a significant decreasing trend in SAVR utilization (*p* = 0.026). A significant steady upward trend was observed for TAVR procedures from 2011 to 2017 with an additional significant elevation after 2014 (*p* = 0.006, Figure 1).

### Clinical Outcomes

**Figure 2:**
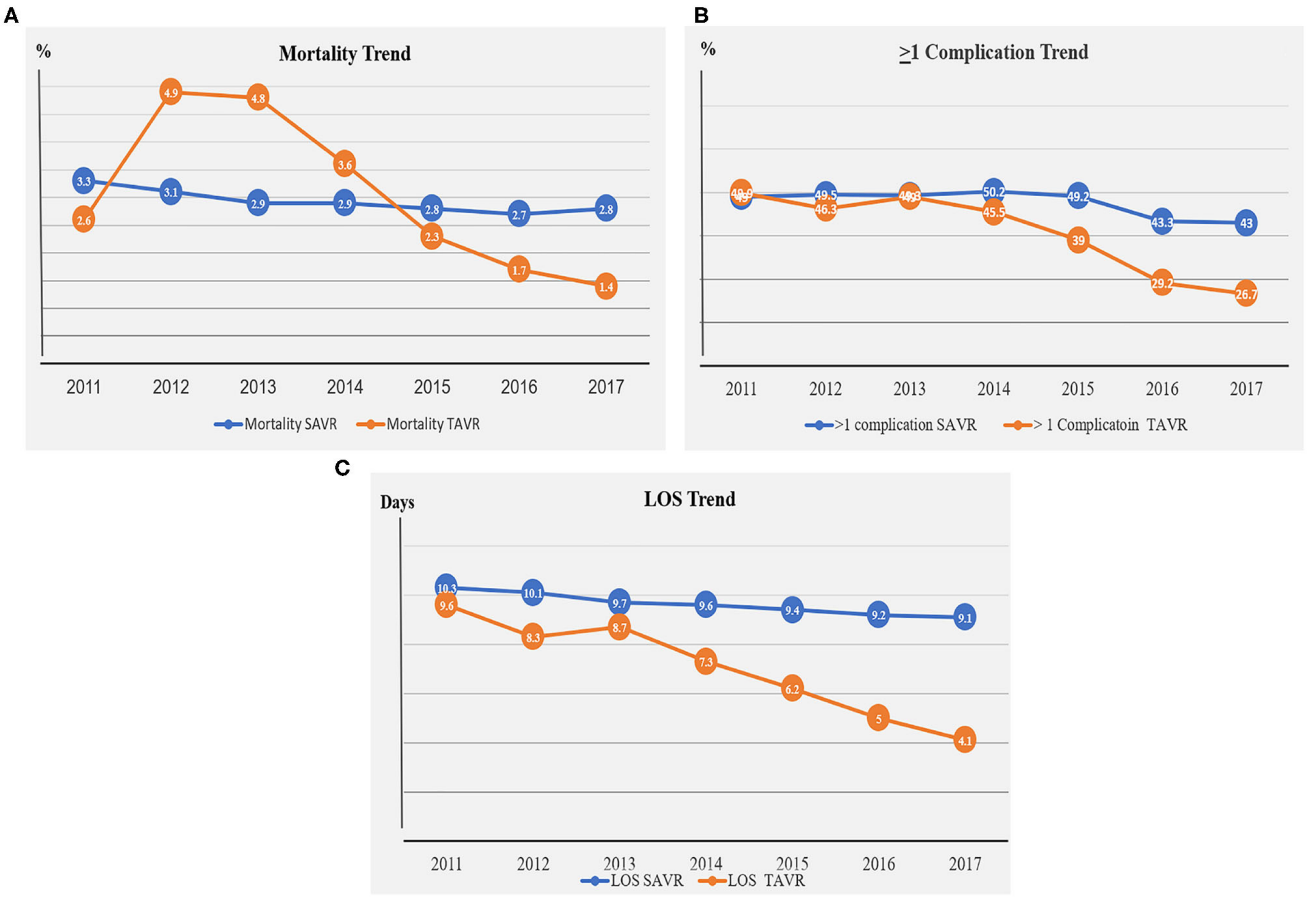
**(A)** Mortality trend in the SAVR and TAVR groups during the years 2011–2017. **(B)** Complication trend in the SAVR and TAVR groups during the years 2011–2017. **(C)** LOS trend in the SAVR and TAVR groups during the years 2011–2017.

The rate of all-cause in-hospital mortality during the study period was 2.9% in the SAVR group compared with 2.3% in the TAVR group, *p* < 0.001 (Table 2). Although in-hospital mortality remained essentially unchanged in the SAVR group throughout the study period, the in-hospital mortality rate decreased about 50% in the TAVR group between the years 2011 and 2017 (3.3–2.8% vs. 2.6–1.4%, respectively; Figure 2A, Table A3).

**Table 2:** Comparison of mortality and complications of SAVR vs. TAVR during the years 2011–2017.

	**SAVR total**	**TAVR total**	***P*-value**
Mortality	2.9	2.3	<0.001
At least one complication	48	34.7	<0.001
Pericardial	4.1	2.7	<0.001
Cardiac	14.3	9.0	<0.001
Pulmonary	12.9	5.1	<0.001
Hemorrhage/hematoma	2.8	1.4	<0.001
Vascular	4.8	4.3	0.005
Infection	4.6	2.1	<0.001
Neurological	1.4	0.9	<0.001
Acute renal failure	17.4	12.4	<0.001
Cardiogenic shock	4.5	2.3	<0.001
Diaphragmatic paralysis	0.1	0.1	0.955
Reopen	2.3	0.2	<0.001
Mechanical complication device related	2.7	2.3	<0.001
Pacemaker	5.4	9.9	<0.001
PVL	0.8	0.9	0.246
Length of stay (days), %	9.7 ± 0.1	5.7 ± 0.1	0.001

*AVR, aortic valve replacement; PVL, paravalvular leak; SAVR, surgical aortic valve replacement; TAVR, transcatheter aortic valve replacement*.

**Figure 3:**
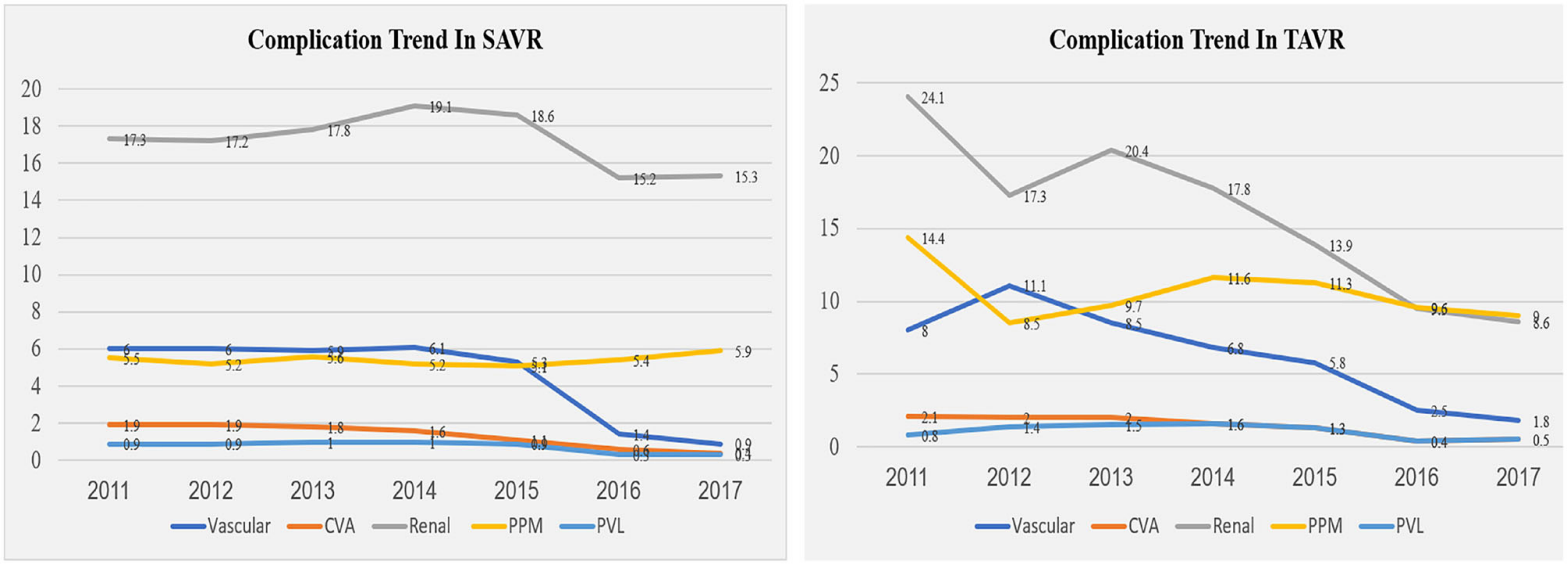
Complication trend in the SAVR and TAVR groups during the years 2011–2017.

At least one peri-procedural complication following the AVR procedures was noted in 48% of the SAVR patients and in 35% of the TAVR patients (Figure 2B, Table A3). Since 2014, downward trends for complications were observed in the SAVR and TAVR groups. A more significant decrease in complication rate was noted in the TAVR group (Figure 2B, Table A3). The leading causes of complication in the SAVR and TAVR groups were renal, cardiac, pulmonary vascular, and PPM (Table 2, Table A3). Two leading complication causes, vascular and renal, decreased significantly during the study period in both groups (Figure 3, Table A3).

LOS was significantly lower in the TAVR group with a mean ± SD of 5.7 ± 0.1 vs. 9.7 ± 0.1 in the SAVR group (*p* = 0.0001, Figure 2C, Table A3). LOS days in the TAVR group decreased significantly from 9.6 days in 2011 to 4.1 days in 2017, whereas in the SAVR group, no significant change was observed during these years, 10.3 in 2011 to 9.1 in 2017 (Figure 2C, Table A3).

### Univariate and Multivariate Analyses

In an unadjusted analysis, we found the following variables to be associated with increased in-hospital mortality for both SAVR and TAVR patients: older age, female sex, chronic obstructive pulmonary disease, renal failure, peripheral vascular disease, and increasing Deyo-CCI score (Table A4). Of note, congestive heart failure and prior cardiac surgery were found to be strongly associated with increased mortality rates only for patients who underwent SAVR. In the early years 2011–2014, all comorbidities were found to be associated with increased mortality rates only for patients who underwent TAVR (*p* < 0.001) (Table A4).

The multivariable regression model analysis adjusted for potential confounders is presented in Table 3. Various individual comorbidities as well as Deyo-CCI index scores remained independent predictors of in-hospital mortality for TAVR and SAVR groups. Patients older than 70 years with prior cerebrovascular disease, renal disease, congestive heart failure, or Deyo-CCI score above 2 are at higher risk for mortality in the SAVR procedure compared with the TAVR procedure. Prior cardiac surgery remained a strong independent predictor of in-hospital mortality in the SAVR patients (Table 2).

**Table 3:** Multivariate analysis for predictors of mortality from 2011 to 2017 in SAVR and TAVR cohort.

	**SAVR**		**TAVR**	
**Predictor**	**Odds ratio (95% CI)**	***P*-value**	**Odds ratio (95% CI)**	***P*-value**
Age group		<0.001		<0.001
18–49 yrs	1.00 (reference)	N/A	1.00 (reference)	N/A
50–59 yrs	1.12 (1.00, 1.26)	0.041	1.90 (0.97, 3.72)	0.060
60–69 yrs	1.13 (1.02, 1.25)	0.019	1.27 (0.67, 2.42)	0.460
70–79 yrs	1.69 (1.53, 1.86)	<0.001	1.34 (0.71, 2.52)	0.362
80–89 yrs	2.28 (2.06, 2.51)	<0.001	1.51 (0.80, 2.82)	0.202
90 yrs or older	3.64 (3.05, 4.33)	<0.001	2.11 (1.12, 3.97)	0.020
Gender		<0.001		<0.001
Male	1.00 (reference)	N/A	1.00 (reference)	N/A
Female	1.38 (1.33, 1.43)	<0.001	1.26 (1.18, 1.35)	<0.001
Race		<0.001		<0.001
White	1.00 (reference)	N/A	1.00 (reference)	N/A
Asian or Pacific Islander	1.34 (1.16, 1.55)	<0.001	0.94 (0.67, 1.32)	0.718
Black	1.57 (1.45, 1.70)	<0.001	0.59 (0.47, 0.74)	<0.001
Hispanic	1.26 (1.17, 1.36)	<0.001	1.32 (1.13, 1.54)	<0.001
Native American	2.35 (1.86, 2.98)	<0.001	2.11 (1.25, 3.57)	0.005
**Comorbidities**
Hypertension		<0.001		<0.001
No	1.00 (reference)	N/A	1.00 (reference)	N/A
Yes	0.59 (0.56,0.61)	<0.001	0.49 (0.46, 0.53)	<0.001
Hyperlipidemia		<0.001		<0.001
No	1.00 (reference)	N/A	1.00 (reference)	N/A
Yes	0.50 (0.49, 0.52)	<0.001	0.48 (0.45, 0.51)	<0.001
Cerebro vascular disease		<0.001		0.058
No	1.00 (reference)	N/A	1.00 (reference)	N/A
Yes	1.79 (1.69, 1.90)	<0.001	1.13 (1.00, 1.28)	0.058
Congestive heart failure		<0.001		0.025
No	1.00 (reference)	N/A	1.00 (reference)	N/A
Yes	3.07 (2.89,3.26)	<0.001	1.10 (1.01,1.20)	0.025
Diabetes Mellitus		<0.001		<0.001
No	1.00 (reference)	N/A	1.00 (reference)	N/A
Yes	1.08 (1.04, 1.12)	<0.001	0.72 (0.66, 0.77)	<0.001
Renal failure		<0.001		<0.001
No	1.00 (reference)	N/A	1.00 (reference)	N/A
Yes	2.14 (2.05, 2.23)	<0.001	1.47 (1.37, 1.57)	<0.001
Chronic pulmonary disease		<0.001		<0.001
No	1.00 (reference)	N/A	1.00 (reference)	N/A
Yes	1.12 (1.07, 1.17)	<0.001	1.17 (1.09, 1.26)	<0.001
Smoker		<0.001		0.302
No	1.00 (reference)	N/A	1.00 (reference)	N/A
Yes	0.77 (0.71, 0.85)	<0.001	1.14 (0.89, 1.45)	0.302
Peripheral vascular disorders		<0.001		<0.001
No	1.00 (reference)	N/A	1.00 (reference)	N/A
Yes	1.44 (1.38, 1.50)	<0.001	1.30 (1.21, 1.39)	<0.001
Prior IHD		<0.001		<0.001
No	1.00 (reference)	N/A	1.00 (reference)	N/A
Yes	1.19 (1.13, 1.24)	<0.001	0.81 (0.74,0.89)	<0.001
Prior PCI		<0.001		<0.001
No	1.00 (reference)	N/A	1.00 (reference)	N/A
Yes	0.76 (0.70, 0.83)	<0.001	0.59 (0.52, 0.67)	<0.001
Prior cardiac surgery		<0.001		<0.001
No	1.00 (reference)	N/A	1.00 (reference)	N/A
Yes	1.28 (1.20, 1.36)	<0.001	0.61 (0.56, 0.67)	<0.001
Isolated surgery		<0.001		<0.001
No	1.00 (reference)	N/A	1.00 (reference)	N/A
Yes	0.42 (0.40, 0.44)	<0.001	0.62 (0.51, 0.76)	<0.001
Deyo-Charlson Comorbidity Index		<0.001		
0	1.00 (reference)	<0.001	1.00 (reference)	N/A
1	1.47 (1.36, 1.58)	N/A	1.09 (0.91, 1.32)	0.354
2 or higher	3.08 (2.9, 3.28)	<0.001	1.49 (1.28, 1.74)	<0.001
Year of procedure		<0.001		<0.001
2011	1.00 (reference)	N/A	1.00 (reference)	N/A
2012	0.96 (0.90, 1.03)	0.231	1.95 (1.33, 2.87)	<0.001
2013	0.89 (0.83, 0.95)	<0.001	1.93 (1.32, 2.82)	<0.001
2014	0.93 (0.87, 0.99)	0.023	1.43 (0.98, 2.09)	0.065
2015	0.89 (0.84, 0.96)	<0.001	0.91 (0.63, 1.34)	0.645
2016	0.87 (0.81, 0.94)	<0.001	0.66 (0.45, 0.97)	0.035
2017	0.94 (0.87, 1.01)	0.104	0.55 (0.38, 0.81)	0.002

*AVR, aortic valve replacement; SAVR, surgical aortic valve replacement; TAVR, transcatheter aortic valve replacement; IHD, ischemic heart disease; PCI, percutaneous coronary intervention*.

In the TAVR group, procedures done during the year 2011–2014 predicted higher mortality compared with procedures done during the years 2015–2017. This is in contrast to the SAVR group that none of the study years was predictive of in-hospital mortality (Table 3).

## Discussion

Utilizing data from the NIS, the largest all-payer inpatient database in the United States, we identified a weighted total of 542,734 patients who underwent AVR during their hospitalization for AS. Our data shows a relatively steady trend in utilization of SAVR in AS patients during the early TAVR era (2011 to 2014) with a significant downward trend in the following years, 2015 to 2017. In contrast, a steady upward trend in TAVR utilization was observed from 2011 with a significant upward trend in the late TAVR era between the years 2015 and 2017.

Prior studies demonstrate the nationwide growth in TAVR volume and penetration rate. Despite the growth in TAVR demand, available data suggests that TAVR has remained relatively underutilized based on estimates of TAVR penetration in Europe and North America (5, 16). Reported penetration rates in the United States and Canada have been low compared with Western European countries due to different regulatory requirements that delayed market access (5); however, these have likely improved over time.

A recent study evaluated the trends in AVR during the years 2003 to 2016 and demonstrated that, in the elderly population of over 60 years, SAVR initially increased gradually from 2003 to 2011 but later declined in 2016. The proportion of TAVR procedures increased remarkably from 2012 to 2016 (26). Our study included all AVR procedures in patients 18 years and older with severe AS, who underwent AVR during their hospitalization since 2011, and the FDA first gave the approval for device use based on the PARTNER trial results (3, 5) until 2017.

We evaluated SAVR trends via two TAVR eras. First, the early TAVR era is between the years 2011 and 2014, a time that was needed for diffusion of a new device in the marketplace. During these years, TAVR penetration was dependent upon the sharing of the SAVR market and then replaced SAVR. Second, the late TAVR era, during the years 2015 to 2017, the TAVR device was adopted as a standard of care in the clinical guidelines.

Our data shows a relatively steady trend in the utilization of SAVR in AS patients during the early TAVR era with a significantly downward trend in the following years. In contrast, a steady upward trend in TAVR utilization was observed from 2011 with a significant upward trend in the late TAVR era. We may argue that the rapid expansion of TAVR is due to robust clinical evidence derived from randomized control trials and large-scale national and international registries, and in many nations, the volume of TAVR now exceeds SAVR in the late era.

Our study reveals the increasing prevalence of older patients and individual comorbidities as well as patients with Deyo-CCI Comorbidity Index > 2 in the TAVR group compared with the SAVR group; both factors are independent predictors of mortality. Despite the higher risk profile in the TAVR group complication rate, the mortality rates were lower in the TAVR group compared with the SAVR group.

The data shows that, although complication rates decreased significantly during the study period in the TAVR group, no significant improvements were observed in the SAVR group as we showed an independent predictor of mortality in the early compared with the late TAVR era. This could be explained by the learning curve of new technology that entered the market.

This topic has become of increasing importance after recent publications showing a clear inverse relationship between TAVR volume center and outcomes of mortality (27–29), similar to that previously demonstrated for patients undergoing other surgical cardiac interventions (30, 31). This could be explained by the small changes in the SAVR field that during these years was already a well implanted technology in contrast to the TAVR technology.

Our study should be interpreted in the contexts of several limitations. First, the NIS database is a retrospective administrative database that contains discharge-level records and, as such, is susceptible to coding errors, and reporting may not be consistent across different institutions. Second, the NIS does not include detailed clinical information and, therefore, cannot rule out residual confounding of the associations we observed. Additionally, the NIS precludes using follow-up beyond the same index hospitalization. These limitations are counterbalanced by the real world, nationwide nature of the data as well as mitigation of reporting bias introduced by selective publication of results from specialized centers.

In conclusion, TAVR was approved in the United States in late 2011, providing a critically needed alternative therapy. The utilization of SAVR in patients with AS remained steady until 2014 and decreased afterward. In contrast, TAVR utilization showed a steady upward trend during the study period. Our study reveals the rising prevalence of comorbidities in patients that require TAVR compared with SAVR. Higher complication and mortality rates were recorded in the SAVR group. Complications and mortality rate were decreased significantly in the TAVR group while comparing the early vs. late years of TAVR use.

## Data Availability Statement

The original contributions presented in the study are included in the article/Supplementary Material, further inquiries can be directed to the corresponding author/s.

## Ethics Statement

The studies involving human participants were reviewed and approved by The NIS database includes only de-identified data; therefore, this study was deemed exempt from institutional review by the Human Research Committee. Written informed consent for participation was not required for this study in accordance with the national legislation and the institutional requirements.

## Author Contributions

GE-G and GR: conceived the idea and design of the study and draft the paper. FK and IM: provided revisions to the manuscript. SC: data analysis and interpretation and provided revisions to the manuscript. DK and HW: data interpretation and provided revision to the manuscript. RA and DP: provided revision to the manuscript. OA: conceived the idea and design of the study and principle investigator. All authors contributed to the article and approved the submitted version.

## Conflict of Interest

The authors declare that the research was conducted in the absence of any commercial or financial relationships that could be construed as a potential conflict of interest.

## Abbreviations

SAVR: Surgical Aortic Valve Replacement
TAVR: Transcatheter Aortic Valve Replacement
AVR: Aortic Valve Replacement.
