# Supplementary material for: Comparing Trajectory of Surgical Aortic Valve Replacement in the Early vs. Late Transcatheter Aortic Valve Replacement Era
Source: Front Cardiovasc Med. 2021 Jun 22;8:680123. doi: 10.3389/fcvm.2021.680123 (PMC8258156; doi:10.3389/fcvm.2021.680123)
Supplement: Supplementary file 1 [file Data_Sheet_1.docx]

**Appendix**

Appendix 1: Deyo-CCI score, ICD-9-CM and ICD-10-CM codes

| **ICD-9 CM codes** | **Condition** | **Score** |
| --- | --- | --- |
| 410 – 410.9 | Myocardial infarction | 1 |
| 428 – 428.9 | Congestive heart failure | 1 |
| 433.9, 441 – 441.9, 785.4, V43.4 | Peripheral vascular disease | 1 |
| 430 – 438 | Cerebrovascular disease | 1 |
| 290 – 290.9 | Dementia | 1 |
| 490 – 496, 500 – 505, 506.4 | Chronic pulmonary disease | 1 |
| 710.0, 710.1, 710.4, 714.0 – 714.2, 714.81, 725 | Rheumatologic disease | 1 |
| 531 – 534.9 | Peptic ulcer disease | 1 |
| 571.2, 571.5, 571.6, 571.4 – 571.49 | Mild liver disease | 1 |
| 250 – 250.3, 250.7 | Diabetes | 1 |
| 250.4 – 250.6 | Diabetes with chronic complications | 2 |
| 344.1, 342 – 342.9 | Hemiplegia or paraplegia | 2 |
| 582 – 582.9, 583 – 583.7, 585, 586, 588 – 588.9 | Renal disease | 2 |
| 140-172.9, 174-195.8, 200-208.9 | Any malignancy including leukemia and lymphoma | 2 |
| 572.2 – 572.8 | Moderate or severe liver disease | 3 |
| 196-199.1 | Metastatic solid tumor | 6 |
| 042 – 044.9 | Acquired Immunodeficiency syndrome (AIDS) | 6 |

| **ICD-10 CM codes** | **Condition** | **Score** |
| --- | --- | --- |
| I21.x, I22.x, I25.2 | Myocardial infarction | 1 |
| I09.9, I11.0, I13.0, I13.2, I25.5, I42.0, I42.5-I42.9, I43.x, I50.x, P29.0 | Congestive heart failure | 1 |
| I70.x, I71.x, I73.1, I73.8, I73.9, I77.1, I79.0,  I79.2, K55.1, K55.8, K55.9, Z95.8, Z95.9 | Peripheral vascular disease | 1 |
| G45.x, G46.x, H34.0, I60.x-I69.x | Cerebrovascular disease | 1 |
| F00.x-F03.x, F05.1, G30.x, G31.1 | Dementia | 1 |
| I27.8, I27.9, J40.x-J47.x, J60.x-J67.x, J68.4, J70.1, J70.3 | Chronic pulmonary disease | 1 |
| M05.x, M06.x, M31.5, M32.x-M34.x, M35.1, M35.3, M36.0 | Rheumatologic disease | 1 |
| K25.x-K28.x | Peptic ulcer disease | 1 |
| B18.x, K70.0-K70.3, K70.9, K71.3-K71.5, K71.7, K73.x, K74.x, K76.0, K76.2-K76.4, K76.8, K76.9, Z94.4 | Mild liver disease | 1 |
| E10.0, E10.l, E10.6, E10.8, E10.9, E11.0, E11.1, E11.6, E11.8, E11.9, E12.0, E12.1, E12.6, E12.8, E12.9, E13.0, E13.1, E13.6, E13.8, E13.9, E14.0, E14.1, E14.6, E14.8, E14.9 | Diabetes | 1 |
| E10.2-E10.5, E10.7, E11.2-E11.5, E11.7, E12.2-E12.5, E12.7, E13.2-E13.5, E13.7, E14.2-E14.5, E14.7 | Diabetes with chronic complications | 2 |
| G04.1, G11.4, G80.1, G80.2, G81.x, G82.x, G83.0-G83.4, G83.9 | Hemiplegia or paraplegia | 2 |
| I12.0, I13.1, N03.2-N03.7, N05.2-N05.7, N18.x, N19.x, N25.0, Z49.0-Z49.2, Z94.0, Z99.2 | Renal disease | 2 |
| C00.x-C26.x, C30.x-C34.x, C37.x-C41.x, C43.x, C45.x-C58.x, C60.x-C76.x, C81.x-C85.x, C88.x, C90.x-C97.x | Any malignancy including leukemia and lymphoma | 2 |
| I85.0, I85.9, I86.4, I98.2, K70.4, K71.1, K72.1, K72.9, K76.5, K76.6, K76.7 | Moderate or severe liver disease | 3 |
| C77.x-C80.x | Metastatic solid tumor | 6 |
| B20.x-B22.x, B24.x | Acquired Immunodeficiency syndrome (AIDS) | 6 |

Appendix 2: In-hospital complication codes for ICD-9-CM and ICD-10-CM

| **Complication** | **ICD-9-CM Code(s)** | **ICD-10-CM Code(s)** |
| --- | --- | --- |
| **Pericardial** |  |  |
| Hemopericardium | 423.0 | I31.2 |
| Tamponade | 423.3 | [I31.4](https://www.icd10data.com/ICD10CM/Codes/I00-I99/I30-I52/I31-/I31.4) |
| Pericardiocentesis | 37.0 | OW9DXXX, OW9CXXX, 0W9D40Z |
| Acute pericarditis | 420.9 | I30.1, I30.8, I30.9 |
| **Cardiac**  (including postoperative cardiac block, myocardial infarction,  cardiac arrest, and congestive heart failure) | 997.1 | I97.710, I97.110, I97.120, I97.130, I97.190, I97.88, I97.89, I97.711, I97.790  I97.XXX |
| **Pulmonary** |  |  |
| Pneumothorax/hemothorax | 512.1-512.2, 511.8 | [J95.811](https://www.icd10data.com/ICD10CM/Codes/J00-J99/J95-J95/J95-/J95.811), [J95.812](https://www.icd10data.com/ICD10CM/Codes/J00-J99/J95-J95/J95-/J95.812), J95.830, J95.831, J94.2 |
| Diaphragm paralysis | 519.4 | [J98.6](https://www.icd10data.com/ICD10CM/Codes/J00-J99/J96-J99/J98-/J98.6) |
| Post-operative Respiratory Failure | 518.51, 518.53 | [J95.821](https://www.icd10data.com/ICD10CM/Codes/J00-J99/J95-J95/J95-/J95.821), [J96.00](https://www.icd10data.com/ICD10CM/Codes/J00-J99/J96-J99/J96-/J96.00), [J95.822](https://www.icd10data.com/ICD10CM/Codes/J00-J99/J95-J95/J95-/J95.822), [J96.20](https://www.icd10data.com/ICD10CM/Codes/J00-J99/J96-J99/J96-/J96.20) |
| Other iatrogenic Respiratory Complications | 997.3 | J95.88, J95.89, J95.851, J95.859 |
| **Hemorrhage/Hematoma** |  |  |
| Hemorrhage/hematoma complicating a procedure | 998.11-998.12 | [I97.411](https://www.icd10data.com/ICD10CM/Codes/I00-I99/I95-I99/I97-/I97.411), [I97.418](https://www.icd10data.com/ICD10CM/Codes/I00-I99/I95-I99/I97-/I97.418), [I97.42](https://www.icd10data.com/ICD10CM/Codes/I00-I99/I95-I99/I97-/I97.42), [I97.611](https://www.icd10data.com/ICD10CM/Codes/I00-I99/I95-I99/I97-/I97.611)  [I97.618](https://www.icd10data.com/ICD10CM/Codes/I00-I99/I95-I99/I97-/I97.618), [I97.620](https://www.icd10data.com/ICD10CM/Codes/I00-I99/I95-I99/I97-/I97.620), [I97.411](https://www.icd10data.com/ICD10CM/Codes/I00-I99/I95-I99/I97-/I97.411), [I97.418](https://www.icd10data.com/ICD10CM/Codes/I00-I99/I95-I99/I97-/I97.418), [I97.42](https://www.icd10data.com/ICD10CM/Codes/I00-I99/I95-I99/I97-/I97.42), [I97.621](https://www.icd10data.com/ICD10CM/Codes/I00-I99/I95-I99/I97-/I97.621), [I97.631](https://www.icd10data.com/ICD10CM/Codes/I00-I99/I95-I99/I97-/I97.631), [I97.638](https://www.icd10data.com/ICD10CM/Codes/I00-I99/I95-I99/I97-/I97.638) |
| Acute post-hemorrhagic anemia | 285.1 | D62 |
| Hemorrhage requiring transfusion | (998.11-998.12, 285.1) AND (99.00-99.09) | [I97.411](https://www.icd10data.com/ICD10CM/Codes/I00-I99/I95-I99/I97-/I97.411), [I97.418](https://www.icd10data.com/ICD10CM/Codes/I00-I99/I95-I99/I97-/I97.418), [I97.42](https://www.icd10data.com/ICD10CM/Codes/I00-I99/I95-I99/I97-/I97.42), [I97.611](https://www.icd10data.com/ICD10CM/Codes/I00-I99/I95-I99/I97-/I97.611)  [I97.618](https://www.icd10data.com/ICD10CM/Codes/I00-I99/I95-I99/I97-/I97.618), [I97.620](https://www.icd10data.com/ICD10CM/Codes/I00-I99/I95-I99/I97-/I97.620), [I97.411](https://www.icd10data.com/ICD10CM/Codes/I00-I99/I95-I99/I97-/I97.411), [I97.418](https://www.icd10data.com/ICD10CM/Codes/I00-I99/I95-I99/I97-/I97.418), [I97.42](https://www.icd10data.com/ICD10CM/Codes/I00-I99/I95-I99/I97-/I97.42), [I97.621](https://www.icd10data.com/ICD10CM/Codes/I00-I99/I95-I99/I97-/I97.621), [I97.631](https://www.icd10data.com/ICD10CM/Codes/I00-I99/I95-I99/I97-/I97.631), [I97.638](https://www.icd10data.com/ICD10CM/Codes/I00-I99/I95-I99/I97-/I97.638)  AND 3023XXX, 3024XXX  30233H0, 30233N0, 30243H0, 30243N0, 30233H1, 30243H1, 30233W0, 30243W0, 30233N1, 30233P1, 30243P1 |
| **Vascular** |  |  |
| Accidental puncture or laceration during a procedure | 998.2, e8700-e709 | [I97.51](https://www.icd10data.com/ICD10CM/Codes/I00-I99/I95-I99/I97-/I97.51), [I97.52](https://www.icd10data.com/ICD10CM/Codes/I00-I99/I95-I99/I97-/I97.52) |
| Injury to blood vessels | 900-904 | S25.X, S35.X |
| Arteriovenous Fistula | 447.0 | I77.0 |
| Injury to retroperitoneum | 868.04 | [S36.899A](https://www.icd10data.com/ICD10CM/Codes/S00-T88/S30-S39/S36-/S36.899A) |
| Vascular complication requiring surgical/Percutaneous repair | 39.30, 39.31, 39.32, 39.41, 39.49, 39.52, 39.53, 39.56, 39.59, 39.90, 39.91, 39.98, 39.99 | [03QY0ZZ](https://www.icd10data.com/ICD10PCS/Codes/0/3/Q/Y/03QY0ZZ), [03QY3ZZ](https://www.icd10data.com/ICD10PCS/Codes/0/3/Q/Y/03QY3ZZ), [03QY4ZZ](https://www.icd10data.com/ICD10PCS/Codes/0/3/Q/Y/03QY4ZZ)  [04QY0ZZ](https://www.icd10data.com/ICD10PCS/Codes/0/4/Q/Y/04QY0ZZ), [04QY3ZZ](https://www.icd10data.com/ICD10PCS/Codes/0/4/Q/Y/04QY3ZZ), [04QY4ZZ](https://www.icd10data.com/ICD10PCS/Codes/0/4/Q/Y/04QY4ZZ)  [05QY0ZZ](https://www.icd10data.com/ICD10PCS/Codes/0/5/Q/Y/05QY0ZZ), [05QY3ZZ](https://www.icd10data.com/ICD10PCS/Codes/0/5/Q/Y/05QY3ZZ), [05QY4ZZ](https://www.icd10data.com/ICD10PCS/Codes/0/5/Q/Y/05QY4ZZ)  [06QY0ZZ](https://www.icd10data.com/ICD10PCS/Codes/0/6/Q/Y/06QY0ZZ), [06QY3ZZ](https://www.icd10data.com/ICD10PCS/Codes/0/6/Q/Y/06QY3ZZ), [06QY4ZZ](https://www.icd10data.com/ICD10PCS/Codes/0/6/Q/Y/06QY4ZZ)  [02QW0ZZ](https://www.icd10data.com/ICD10PCS/Codes/0/2/Q/W/02QW0ZZ), [02QW3ZZ](https://www.icd10data.com/ICD10PCS/Codes/0/2/Q/W/02QW3ZZ), [02QX4ZZ](https://www.icd10data.com/ICD10PCS/Codes/0/2/Q/X/02QX4ZZ)  [03Q00ZZ](https://www.icd10data.com/ICD10PCS/Codes/0/3/Q/0/03Q00ZZ), [03Q03ZZ](https://www.icd10data.com/ICD10PCS/Codes/0/3/Q/0/03Q03ZZ), 03Q04ZZ,  03Q10ZZ, 03Q13ZZ, 03Q14ZZ, 03Q20ZZ, 03Q23ZZ, 03Q24ZZ, 03Q30ZZ, 03Q40ZZ, 03Q33ZZ, 03Q43ZZ, 03Q44ZZ, 03Q50ZZ, 03Q53ZZ, 03Q54ZZ, 03Q60ZZ, 03Q63ZZ, 03Q64ZZ, 03Q74ZZ 03Q70ZZ, 03Q73ZZ, 03Q80ZZ, 03Q83ZZ, 03Q84ZZ, 03Q90ZZ, 03Q93ZZ, 03Q94ZZ, 03QA0ZZ, 03QA3ZZ, 03QA4ZZ, 03QB0ZZ, 03QB3ZZ, 03QB4ZZ,03QC0ZZ, 03QC3ZZ, 03QC4ZZ,03QY0ZZ, 03QY3ZZ,03QY4ZZ,04Q00ZZ,  04Q03ZZ, 04QC0ZZ, 04QC3ZZ  04Q04ZZ, 04QC4ZZ,04QD0ZZ, 04QD3ZZ,04QD4ZZ, 04QE0ZZ 04QE3ZZ, 04QE4ZZ, 04QF0ZZ, 04QF3ZZ, 04QF4ZZ, 04QH0ZZ, 04QH3ZZ, 04QH4ZZ,04QJ0ZZ  04QJ3ZZ, 04QJ4ZZ,04QK0ZZ, 04QK3ZZ, 04QL0ZZ, 04QL3ZZ, 04QL4ZZ, 04QY0ZZ, 04QY3ZZ |
| Other vascular complications | 997.2, 997.7 | [T81.719A](https://www.icd10data.com/ICD10CM/Codes/S00-T88/T80-T88/T81-/T81.719A) , [T81.72XA](https://www.icd10data.com/ICD10CM/Codes/S00-T88/T80-T88/T81-/T81.72XA)  T82.837, T82.838 |
| **Infection** |  |  |
| Fever | 780.60, 780.62 | T82.6, T82.7, R50.82 |
| Septicemia | 038.*, 995.91-995.92, 998.02, 790.7 | A41.9, A65.20, [T81.12XA](https://icd.codes/icd10cm/T8112XA) |
| Post-procedural aspiration pneumonia | 997.32 | J95.89 |
| **Neurological** |  |  |
| Nervous system complication, unspecified | 997.00 | [G97.81](https://www.icd10data.com/ICD10CM/Codes/G00-G99/G89-G99/G97-/G97.81) |
| Central nervous system complication | 997.01 | G97.81, G97.82 |
| Iatrogenic cerebrovascular infarction or hemorrhage | 997.02 | [I97.811](https://www.icd10data.com/ICD10CM/Codes/I00-I99/I95-I99/I97-/I97.811), [I97.821](https://www.icd10data.com/ICD10CM/Codes/I00-I99/I95-I99/I97-/I97.821)  I97.810 |
| Transient ischemic attack | 435.9 | [G45.9](https://www.icd10data.com/ICD10CM/Codes/G00-G99/G40-G47/G45-/G45.9), [I67.848](https://www.icd10data.com/ICD10CM/Codes/I00-I99/I60-I69/I67-/I67.848) |
| Any stroke | 430, 431, 432, 433.01, 433.11, 433.21, | 160.9,161.9, 163.22, 163.139, 163.239  163.019,163.119, 163.219 |
| **Acute renal failure** | 584.5-584.9 | N17, N17.1, N17.2, N17.8, N17.9, N99.0, |
| **Cardiogenic shock** | 785.51 | [R57.0](https://www.icd10data.com/ICD10CM/Codes/R00-R99/R50-R69/R57-/R57.0) |
| **Diaphragmatic Paralysis** | 519.4 | [J98.6](https://www.icd10data.com/ICD10CM/Codes/J00-J99/J96-J99/J98-/J98.6) |
| **Re-open:** | 34.03, 39.41 | 0W39OZZ, OW3BOZZ, OW3COZZ, OW3DOZZ, OW3QOZZ |
| **Mechanical complication device related** |  | T82.01, T82.02, T82.03, T82.09  T82,221, T82.222, T82.223, T82.228  T82.817, T82.818 |
| **PVL** | [996.02](http://www.icd9data.com/2015/Volume1/800-999/996-999/996/996.02.htm?__hstc=93424706.ea137040e43c48c3aaabd4b8d8fc49ab.1570976617663.1586967485270.1587292359526.33&__hssc=93424706.6.1587292359526&__hsfp=2094543708) | [T82.03XA](https://www.icd10data.com/ICD10CM/Codes/S00-T88/T80-T88/T82-/T82.03XA) |
| **Pacemaker implantation** | 37.80-37.83, 37.94, 37.95, 37.96 | OJH606Z, OJH636Z, 0JH806Z, OJH836Z, OJH60PZ, OJH63PZ, 0JH80PZ, OJH83PZ, OJH604Z, OJH634Z, OJH804Z, OJH834Z, OJH605Z, 0JH635Z, OJH805Z, 0JH835Z, 02H73KZ, O2HK3KZ,02HL3KZ,02HN0KZ, 02HN4KZ, OJH608Z, OJH638Z, OJH808Z, 0JH838Z, 02H60KZ, 02H63KZ, 02H64KZ, 02H70KZ, 02H73KZ, 02H74KZ, 02HK0KZ, 02HK3KZ, 02HK4KZ,02HL0KZ, 02HL3KZ, 02HL4KZ, 0JH608Z, 0JH638Z, 0JH808Z, 0JH838Z, 02H60KZ, 02H63KZ, 02H64KZ, 02H70KZ, 02H73KZ, 02H74KZ, 02HK0KZ, 02HK3KZ, 02HK4KZ, 02HL0KZ, 02HL3KZ, 02HL4KZ, 0JH608Z, 0JH638Z,  0JH808Z, 0JH838Z |

Appendix 3: Trends in in-hospital mortality, >1 complication, LOS and complications in SAVR *vs* TAVR groups during the years 2011-2017

|  | **Mortality** | | | **LOS** | | | **At least 1 complication** | | |
| --- | --- | --- | --- | --- | --- | --- | --- | --- | --- |
|  | **SAVR** | **TAVR** | **P-Value** | **SAVR** | **TAVR** | **P-Value** | **SAVR** | **TAVR** | **p-value** |
| **Total** | **2.9** | **2.3** | **<.001** | **9.7+0.1** | **5.7+0.1** | **0.03** | **48** | **34.7** | **<.001** |
| 2011 | 3.3 | 2.6 | 0.49 | 10.3+0.2 | 9.6+0.4 | 0.06 | 49 | 49.9 | 0.7 |
| 2012 | 3.1 | 4.9 | 0.006 | 10.1+0.1 | 8.3+0.2 | 0.001 | 49.5 | 46.3 | 0.04 |
| 2013 | 2.9 | 4.8 | <.001 | 9.7+0.1 | 8.7+0.2 | 0.001 | 49.3 | 49 | 0.82 |
| 2014 | 2.9 | 3.6 | 0.05 | 9.6+0.1 | 7.3+0.2 | 0.001 | 50.2 | 45.5 | <0.001 |
| 2015 | 2.8 | 2.3 | 0.09 | 9.2+0.1 | 6.2+0.1 | 0.001 | 49.2 | 39 | <0.001 |
| 2016 | 2.7 | 1.7 | <0.001 | 9.2+0.1 | 5.0+0.1 | 0.001 | 43.3 | 29.2 | <0.001 |
| 2017 | 2.8 | 1.4 | <0.001 | 9.1+0.1 | 4.1+0.1 | 0.001 | 43 | 26.7 | <0.001 |

|  | **Vascular** | | | **Neurological** | | | **PPM** | | |
| --- | --- | --- | --- | --- | --- | --- | --- | --- | --- |
|  | **SAVR** | **TAVR** | **P-Value** | **SAVR** | **TAVR** | **P-Value** | **SAVR** | **TAVR** | **P-Value** |
| **Total** | **4.8** | **4.3** | **0.005** | **1.4** | **0.9** | **<.001** | **5.4** | **9.9** | **<.001** |
| 2011 | 6 | 8 | 0.34 | 1.9 | 2.1 | 0.86 | 5.5 | 14.4 | 0.003 |
| 2012 | 6 | 11.1 | <.001 | 1.9 | 2 | 0.79 | 5.2 | 8.5 | <.001 |
| 2013 | 5.9 | 8.5 | <.001 | 1.8 | 2 | 0.53 | 5.6 | 9.7 | <.001 |
| 2014 | 6.1 | 6.8 | 0.2 | 1.6 | 1.6 | 0.95 | 5.2 | 11.6 | <.001 |
| 2015 | 5.3 | 5.8 | 0.25 | 1.1 | 1.3 | 0.43 | 5.1 | 11.3 | <.001 |
| 2016 | 1.4 | 2.5 | <.001 | 0.6 | 0.4 | 0.07 | 5.4 | 9.6 | <.001 |
| 2017 | 0.9 | 1.8 | <.001 | 0.4 | 0.5 | 0.88 | 5.9 | 9 | <.001 |

|  | **Acute Renal Failure** | | | **PVL** | | | **Device Related**  **Complication** | | |
| --- | --- | --- | --- | --- | --- | --- | --- | --- | --- |
|  | **SAVR** | **TAVR** | **P-Value** | **SAVR** | **TAVR** | **P-Value** | **SAVR** | **TAVR** | **P-Value** |
| **Total** | **17.4** | **12.4** | **<.001** | **0.8** | **0.9** | **0.25** | **2.7** | **2.3** | **<.001** |
| 2011 | 17.3 | 24.1 | 0.05 | 0.9 | 0.8 | 0.85 | 3.2 | 3.1 | 0.3 |
| 2012 | 17.2 | 17.3 | 0.98 | 0.9 | 1.4 | 0.17 | 3.2 | 3.9 | 0.94 |
| 2013 | 17.8 | 20.4 | 0.009 | 1 | 1.5 | 0.06 | 3.5 | 4.3 | 0.09 |
| 2014 | 19.1 | 17.8 | 0.11 | 1 | 1.6 | 0.01 | 3.6 | 4.7 | 0.005 |
| 2015 | 18.4 | 13.8 | <.001 | 0.9 | 1.3 | 0.05 | 3.1 | 3.9 | 0.014 |
| 2016 | 14.7 | 9.4 | <.001 | 0.3 | 0.4 | 0.08 | 0.6 | 0.7 | 0.5 |
| 2017 | 14.7 | 8.5 | <.001 | 0.3 | 0.5 | 0.11 | 0.7 | 0.8 | 0.4 |

|  | **Pericardial** | | | **Cardiac** | | | **Pulmonary** | | |
| --- | --- | --- | --- | --- | --- | --- | --- | --- | --- |
|  | **SAVR** | **TAVR** | **P-Value** | **SAVR** | **TAVR** | **P-Value** | **SAVR** | **TAVR** | **P-Value** |
| **Total** | **4.1** | **2.7** | **<.001** | **14.3** | **9** | **<.001** | **12.9** | **5.1** | **<.001** |
| 2011 | 4 | 0.8 | 0.004 | 14.3 | 9.4 | 0.005 | 9.6 | 7.7 | 0.43 |
| 2012 | 3.9 | 3.2 | 0.199 | 12.7 | 7.9 | <.001 | 15.8 | 9.1 | <.001 |
| 2013 | 4 | 3.7 | 0.46 | 12.4 | 8.6 | <.001 | 14.7 | 8.5 | 0.003 |
| 2014 | 4 | 2.9 | 0.002 | 14.5 | 8.6 | <.001 | 13.4 | 8.6 | <.001 |
| 2015 | 4.3 | 2.9 | <.001 | 15 | 8.6 | <.001 | 12.6 | 5.3 | <.001 |
| 2016 | 4.2 | 2.5 | <.001 | 16.4 | 9.8 | <.001 | 12.1 | 3.3 | <.001 |
| 2017 | 4.4 | 1.4 | <.001 | 15.9 | 8.9 | <.001 | 11.2 | 2.5 | <.001 |

|  | **Infection** | | | **Hemorrhage** | | | **Cardiogenic Shock** | | | **Re-open/**  **Conversion** | | |  |
| --- | --- | --- | --- | --- | --- | --- | --- | --- | --- | --- | --- | --- | --- |
|  | **SAVR** | **TAVR** | **P-Value** | **SAVR** | **TAVR** | **P-Value** | **SAVR** | **TAVR** | **P-Value** | **SAVR** | **TAVR** | **P-Value** | |
| **Total** | **4.6** | **2.1** | **<.001** | **2.8** | **1.4** | **<.001** | **4.5** | **2.3** | **<.001** | **2.3** | **0.4** | **<.001** | |
| 2011 | 5 | 4.1 | 0.55 | 4 | 4.5 | 0.7 | 4.7 | 5.8 | 0.4 | 3.1 | 0.8 | 0.009 | |
| 2012 | 4.8 | 4.1 | 0.22 | 3.4 | 3.9 | 0.38 | 4 | 3.8 | 0.74 | 2.6 | 0.8 | 0.22 | |
| 2013 | 4.6 | 4.4 | 0.77 | 3.4 | 3.1 | 0.6 | 4.3 | 2.7 | 0.45 | 2.7 | 1.2 | <.001 | |
| 2014 | 4.5 | 3.5 | 0.003 | 3.2 | 2.2 | <.001 | 4.6 | 2.6 | <.001 | 2.3 | 0.7 | <.001 | |
| 2015 | 4.9 | 2.3 | <.001 | 2.4 | 2.1 | 0.001 | 3.1 | 3.9 | <.001 | 2 | 0.7 | <.001 | |
| 2016 | 4.2 | 1.2 | <.001 | 1.3 | 0.7 | 0.001 | 5.2 | 1.9 | <.001 | 1.4 | 0.2 | <.001 | |
| 2017 | 4 | 1.3 | <.001 | 1.4 | 0.5 | <.001 | 5.3 | 1.6 | <.001 | 1.4 | 0.2 | <.001 | |

Table 4: Univariate analysis for predictors of mortality from 2011 to 2017 in SAVR and TAVR cohorts

|  | **SAVR** | | **TAVR** | |
| --- | --- | --- | --- | --- |
| **Predictor** | **Odds Ratio (95% CI)** | **P-Value** | **Odds Ratio (95% CI)** | **P-Value** |
| **Age Group** |  | <.001 |  | <.001 |
| 18-49 yrs | 1.00 (reference) | N/A | 1.00 (reference) | N/A |
| 50-59 yrs | 1.10 (0.98,1.23) | 0.100 | 1.80 (0.92,3.51) | 0.086 |
| 60-69 yrs | 1.10 (0.99,1.22) | 0.069 | 1.14 (0.60,2.16) | 0.688 |
| 70-79 yrs | 1.65 (1.49,1.81) | <.001 | 1.20 (0.64,2.26) | 0.565 |
| 80-89 yrs | 2.24 (2.03,2.47) | <.001 | 1.46 (0.78,2.73) | 0.239 |
| 90 yrs or older | 3.65 (3.06,4.34) | <.001 | 2.15 (1.15,4.04) | 0.017 |
| **Gender** |  | <.001 |  | <.001 |
| Male | 1.00 (reference) | N/A | 1.00 (reference) | N/A |
| Female | 1.46 (1.41,1.52) | <.001 | 1.29 (1.21,1.38) | <.001 |
| **Race** |  | <.001 |  | <.001 |
| White | 1.00 (reference) | N/A | 1.00 (reference) | N/A |
| Asian or Pacific Islander | 1.28 (1.11,1.47) | <.001 | 0.88 (0.63,1.23) | 0.442 |
| Black | 1.44 (1.33,1.56) | <.001 | 0.59 (0.47,0.74) | <.001 |
| Hispanic | 1.18 (1.09,1.27) | <.001 | 1.26 (1.08,1.47) | 0.003 |
| Native American | 2.07 (1.64,2.62) | <.001 | 2.00 (1.19,3.37) | 0.009 |
| ***Comorbidities*** |  |  |  |  |
| **Hypertension** |  | <.001 |  | <.001 |
| No | 1.00 (reference) | N/A | 1.00 (reference) | N/A |
| Yes | 0.65 (0.63,0.68) | <.001 | 0.88 (0.82,0.94) | <.001 |
| **Hyperlipidemia** |  | <.001 |  | <.001 |
| No | 1.00 (reference) | N/A | 1.00 (reference) | N/A |
| Yes | 0.54 (0.52,0.56) | <.001 | 0.47 (0.44,0.50) | <.001 |
| **Cerebro vascular disease** |  | <.001 |  | <.001 |
| No | 1.00 (reference) | N/A | 1.00 (reference) | N/A |
| Yes | 1.90 (1.80,2.01) | <.001 | 1.76 (1.56,1.98) | <.001 |
| **Congestive heart failure** |  | <.001 |  | <.001 |
| No | 1.00 (reference) | N/A | 1.00 (reference) | N/A |
| Yes | 2.55 (2.42,2.69) | <.001 | 0.75 (0.69,0.81) | <.001 |
| **Diabetes Mellitus** |  | <.001 |  | <.001 |
| No | 1.00 (reference) | N/A | 1.00 (reference) | N/A |
| Yes | 1.10 (1.05,1.14) | <.001 | 0.68 (0.63,0.73) | <.001 |
| **Renal failure** |  | <.001 |  | <.001 |
| No | 1.00 (reference) | N/A | 1.00 (reference) | N/A |
| Yes | 2.28 (2.19,2.38) | <.001 | 1.42 (1.32,1.52) | <.001 |
| **Chronic pulmonary disease** |  | <.001 |  | <.001 |
| No | 1.00 (reference) | N/A | 1.00 (reference) | N/A |
| Yes | 1.11 (1.06,1.16) | <.001 | 1.15 (1.08,1.24) | <.001 |
| **Smoker** |  | <.001 |  | <.001 |
| No | 1.00 (reference) | N/A | 1.00 (reference) | N/A |
| Yes | 0.64 (0.59,0.70) | <.001 | 1.63 (1.28,2.07) | <.001 |
| **Peripheral vascular disorders** |  | <.001 |  | <.001 |
| No | 1.00 (reference) | N/A | 1.00 (reference) | N/A |
| Yes | 1.37 (1.32,1.43) | <.001 | 1.33 (1.24,1.42) | <.001 |
| **Prior Ischemic Heart Disease** |  | <.001 |  | <.001 |
| No | 1.00 (reference) | N/A | 1.00 (reference) | N/A |
| Yes | 1.24 (1.19,1.28) | <.001 | 1.66 (1.55,1.78) | <.001 |
| **Prior Percutaneous Coronary Intervention** |  | <.001 |  | 0.011 |
| No | 1.00 (reference) | N/A | 1.00 (reference) | N/A |
| Yes | 0.80 (0.74,0.87) | <.001 | 0.85 (0.76,0.96) | 0.011 |
| **Prior Cardiac surgery** |  | <.001 |  | <.001 |
| No | 1.00 (reference) | N/A | 1.00 (reference) | N/A |
| Yes | 1.24 (1.17,1.32) | <.001 | 0.51 (0.47,0.56) | <.001 |
| **Isolated surgery** |  | <.001 |  | <.001 |
| No | 1.00 (reference) | N/A | 1.00 (reference) | N/A |
| Yes | 0.43 (0.41,0.45) | <.001 | 0.40 (0.38,0.43) | <.001 |
| **Deyo-CCI** | 0.78 (0.75,0.81) | <.001 |  | <.001 |
| 0 | 1.00 (reference) | <.001 | 1.00 (reference) | N/A |
| 1 | 1.45 (1.35,1.56) | N/A | 1.05 (0.87,1.26) | 0.603 |
| 2 or higher | 3.18 (2.99,3.38) | <.001 | 1.33 (1.14,1.55) | <.001 |
| **Year** |  | <.001 |  | <.001 |
| 2011 | 1.00 (reference) | <.001 | 1.00 (reference) | N/A |
| 2012 | 0.95 (0.89,1.01) | N/A | 1.92 (1.31,2.82) | <.001 |
| 2013 | 0.86 (0.80,0.92) | 0.086 | 1.87 (1.28,2.74) | 0.001 |
| 2014 | 0.88 (0.82,0.93) | <.001 | 1.37 (0.94,2.00) | 0.102 |
| 2015 | 0.84 (0.78,0.89) | <.001 | 0.88 (0.60,1.28) | 0.496 |
| 2016 | 0.81 (0.75,0.87) | <.001 | 0.63 (0.43,0.92) | 0.017 |
| 2017 | 0.85 (0.79,0.91) | <.001 | 0.52 (0.35,0.76) | <.001 |

AVR= Aortic Valve Replacement; Deyo-CCI=Deyo-Charlson Comorbidity Index; SAVR= Surgical Aortic Valve Replacement; TAVR= Transcatheter Aortic Valve Replacement
